# Functional Domain Analysis of the Cell Division Inhibitor EzrA

**DOI:** 10.1371/journal.pone.0102616

**Published:** 2014-07-28

**Authors:** Adrian D. Land, Qingwei Luo, Petra Anne Levin

**Affiliations:** Department of Biology, Washington University, Saint Louis, Missouri, United States of America; Loyola University Medical Center, United States of America

## Abstract

The precise spatial and temporal control of bacterial cell division is achieved through the balanced actions of factors that inhibit assembly of the tubulin-like protein FtsZ at aberrant subcellular locations or promote its assembly at the future sites of division. In *Bacillus subtilis*, the membrane anchored cell division protein EzrA, interacts directly with FtsZ to prevent aberrant FtsZ assembly at cell poles and contributes to the inherently dynamic nature of the cytokinetic ring. Recent work suggests EzrA also serves as a scaffolding protein to coordinate lateral growth with cell wall biosynthesis through interactions with a host of proteins, a finding consistent with EzrA's four extensive coiled-coil domains. In a previous study we identified a conserved patch of residues near EzrA's C-terminus (the QNR motif) that are critical for maintenance of a dynamic cytokinetic ring, but dispensable for EzrA-mediated inhibition of FtsZ assembly at cell poles. In an extension of this work, here we report that EzrA's two C-terminal coiled-coils function in concert with the QNR motif to mediate interactions with FtsZ and maintain the dynamic nature of the cytokinetic ring. In contrast, EzrA's two N-terminal coiled-coils are dispensable for interaction between EzrA and FtsZ *in vitro* and *in vivo*, but required for EzrA mediated inhibition of FtsZ assembly at cell poles. Finally, chimeric analysis indicates that EzrA's transmembrane anchor plays a generic role: concentrating EzrA at the plasma membrane where presumably it can most effectively modulate FtsZ assembly.

## Introduction

Assembly of the highly conserved tubulin-like protein FtsZ into a ring structure at the nascent division site initiates the process of cell division in most bacteria. The FtsZ ring serves as a foundation for assembly of the division machinery and constricts at the leading edge of the invaginating septum during cytokinesis. The precise temporal and spatial regulation of cell division is achieved through the actions of a host of proteins, which interact directly with FtsZ to modulate assembly of the cytokinetic ring. Some of these modulators help stabilize FtsZ polymers at midcell and thus maintain the integrity of the cytokinetic ring. In both *Bacillus subtilis* and *Escherichia coli*, the location of FtsZ ring formation appears to be dictated in part through the actions of proteins that inhibit FtsZ assembly at aberrant subcellular positions[1], [2].

In *B. subtilis*, EzrA, a 65 kDa membrane bound protein, plays an important role in both modulatory roles [3], [4]. EzrA is among the first set of proteins to localize to the cytokinetic ring [5]. Null mutations in *ezrA* reduce the critical concentration of FtsZ required for ring formation *in vivo* and result in the formation of extra FtsZ rings and septa at cell poles [3]. In contrast to loss of function mutations in other positional regulators of bacterial cell division, the loss of EzrA significantly increases the stability of the medial FtsZ ring, rendering it resistant to overexpression of division inhibitors [6], [7]. Null mutations in *ezrA* or a point mutation that disrupts EzrA localization to midcell increase cell length by more than 50%, consistent with a model in which EzrA is required for the efficient use of the medial division site. Biochemical experiments indicate that EzrA interacts directly with FtsZ to inhibit assembly [4].

Recent work in both *B. subtilis* and *Staphylococcus aureus* suggest that EzrA may have a second role in which it helps coordinate assembly of the cell division machinery with synthesis of the lateral cell wall. In *B. subtilis* combining a null mutation in *ezrA* with loss of function mutations in *gpsB*, a gene implicated in cell elongation, or *sepF* or *zapA*, both of which play a role in promoting stabilizing lateral interactions between FtsZ protofilaments, severely reduces viability [8]–[10]. Localization studies suggest EzrA functions together with GspB to mediate transfer of the transglycosylase-transpeptidase PBP1 between the lateral and septal cell wall synthesis apparati [8]. EzrA also appears to act coordinately with the essential late stage cell division protein FtsL, to promote constriction of the cytokinetic ring [11]. A null mutation in *ezrA* is synthetic lethal with expression of *yneA*, whose product interacts with FtsL, a downstream component of the division machinery, to block division in response to DNA damage. Intriguingly, the EzrA-YneA synthetic lethal phenotype is suppressed by overexpression of FtsL.

EzrA is also important for coordinating *S. aureus* cell wall synthesis with division and for maintaining cell size [12]. Depleting EzrA leads to disruption of cross wall synthesis (the only mode of growth in this organism) and increased size heterogeneity [12], [13]. *S. aureus* EzrA plays a role in the localization of GpsB and PBP1, as it does in *B. subtilis* [12], [13]. EzrA has been reported to be essential in the rod shaped bacterium, *Listeria monocytogenes*, although its role in cell division and cell wall synthesis has not been well characterized in this organism [14].

Together these data support a model in which EzrA serves as a scaffolding protein, helping to coordinate cell wall synthesis with cell division. Consistent with this idea, several studies have identified interactions between EzrA and proteins involved in both cell division and cell wall synthesis by Bacterial Two-Hybrid analysis (BACTH). BACTH has identified interactions between *B. subtilis* EzrA and FtsA, PBP1, SepF, and GpsB [8], [15]. In *S. aureus* BACTH analysis suggests EzrA interacts directly not only with FtsZ, GpsB, PBP1, and SepF, but also with PBP3, PBP2, DivIB, DivIC, FtsL, and RodA [12], [13]. While it is easy to imagine a scenario in which the cytoplasmic domain of EzrA interacts directly with other primarily cytoplasmic proteins (e.g. FtsA, SepF and GpsB) it is more difficult to explain the apparently direct interactions between EzrA and primarily extracellular proteins (e.g. PBP1, FtsL and DivIC). Only two residues of *B. subtilis* EzrA are predicted to be extracellular and *S. aureus* EzrA does not appear to have any extracellular residues. EzrA's transmembrane helix is poorly conserved at the primary sequence level, suggesting it is unlikely to play a role in mediating conserved protein-protein interactions.

Although primary sequence conservation is limited, EzrAs from a range of bacterial species share a common set of features including an N-terminal membrane anchor, between three and five long coiled-coils, and finally a small patch of highly conserved residues near the C-terminus we have termed the QNR motif [7]. In previous work we determined that the QNR motif is essential for EzrA function in *Bacillus subtilis*. Defects in EzrA's QNR motif disrupt EzrA localization to midcell, stabilizing the medial FtsZ ring and increasing cell length by 50% [7]. Significantly, although QNR mutants are defective in its interaction with FtsZ at midcell, they are still competent to inhibit aberrant FtsZ assembly and division at cell poles. These results suggest EzrA has two genetically separable activities: preventing FtsZ assembly at aberrant subcellular locations and maintaining the dynamic nature of the medial FtsZ ring. The latter function is consistent with EzrA's proposed role in constriction of the FtsZ ring [11].

To clarify EzrA's role as a cell division inhibitor and potential scaffolding protein, we undertook an extensive analysis of the EzrA polypeptide. Our data indicate that while the 69-residue C-terminal domain that includes the QNR motif is sufficient for the robust inhibition of FtsZ assembly *in vitro*, EzrA's transmembrane helix and coiled-coil domains play important yet divergent roles in mediating EzrA's ability to prevent aberrant FtsZ assembly at cell poles and ensures the integrity and dynamic nature of medial FtsZ ring.

## Results

### A 69 residue C-terminal EzrA fragment that includes EzrA's QNR motif is sufficient to inhibit FtsZ assembly *in vitro*

**Figure 1:**
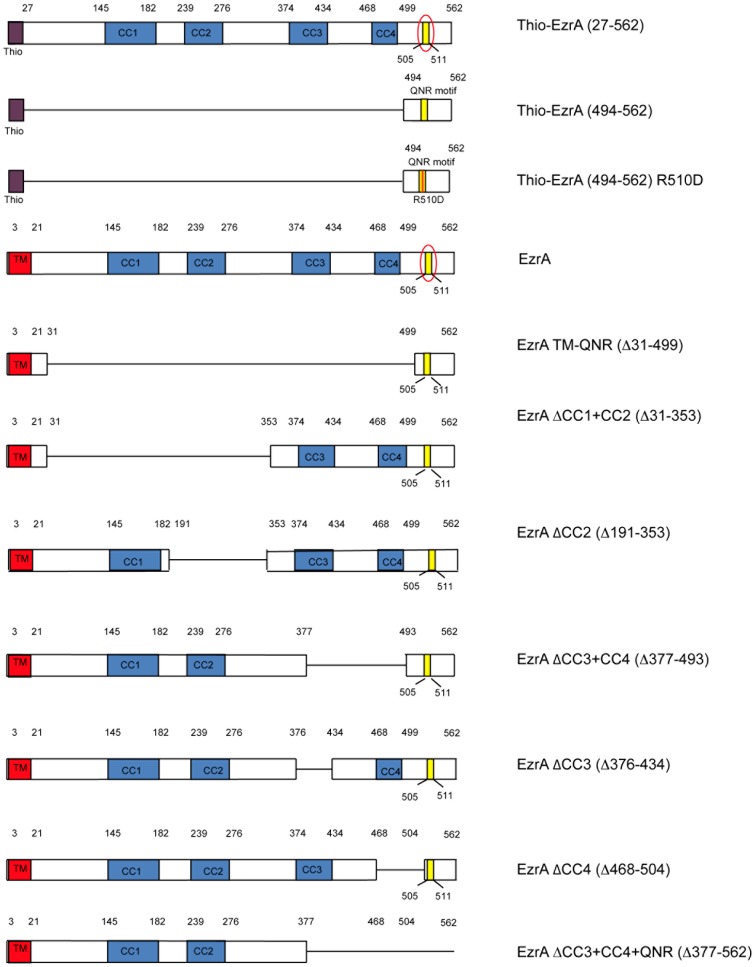
Schematic of EzrA deletion mutants employed in this study. The predicted coiled-coil structure of EzrA is drawn to scale. Numbers refer to amino acid positions. Coiled-coil regions are shaded blue. The QNR motif is shaded yellow and circled in the full-length protein. Lines indicate deleted regions. The QNR domain construct missing all four coiled-coils is just under the full-length protein. All constructs used for *in vivo* analysis were fused in-frame to a C-terminal GFP moiety (not shown) to simplify analysis. All constructs used for *in vitro* analysis were fused in-frame to a N-terminal Thio tag to enhance solubility and a N-terminal 6x-His tag for purification purposes (not shown).

Genetic analysis of EzrA suggests its C-terminus, particularly the QNR motif, is important for maintaining the dynamic nature of the medial FtsZ ring [7]. To determine if the C-terminal region of EzrA that includes the QNR motif is sufficient to inhibit FtsZ assembly on its own, we engineered a protein construct in which an N-terminal thioredoxin tag (to increase solubility) was fused to the last 69 residues of EzrA (494–562). A 6× His tag was also included at the C-terminus for affinity purification purposes (Figure 1). This fusion does not include any of the regions predicted to have significant coiled-coil structure. For comparative purposes, we generated a similar construct that was identical with the exception of a single mutation, R510D, in the QNR motif (Figure 1). In the context of the full length protein, the R510D mutation disrupts EzrA localization to midcell, although the mutant protein retained the ability to inhibit aberrant FtsZ assembly at cell poles [7].

Note that we routinely eliminate EzrA's transmembrane domain for *in vitro* work. While our data (below) support an important role for the TM in concentrating EzrA at the plasma membrane *in vivo*, the continuity of our genetic and biochemical data suggest that removing this domain has little if any impact on the interaction between EzrA and FtsZ.

**Figure 2:**
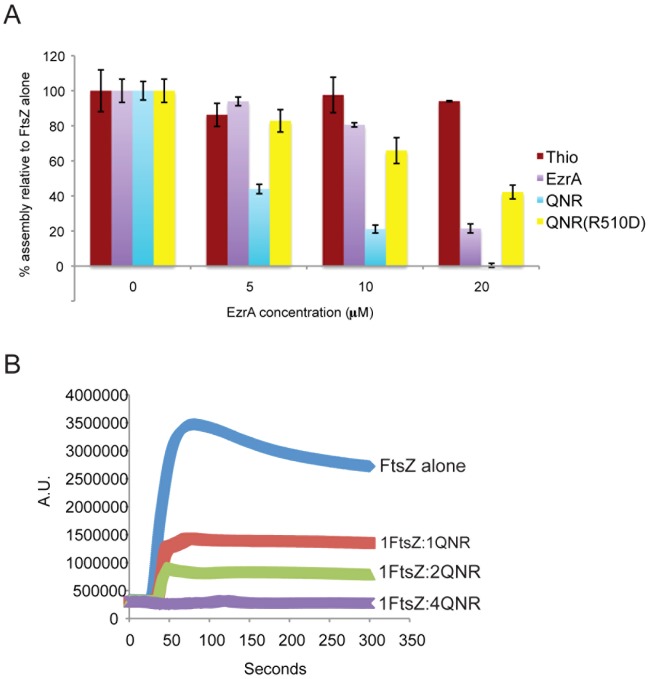
EzrA's QNR domain is sufficient to inhibit FtsZ assembly *in vitro*. (A) Data from 90°-angle light scattering experiments examining the impact of different EzrA constructs on FtsZ assembly *in vitro*. FtsZ is 5 µM in all reactions. Bars represent the maximum scatter averaged across three independent experiments with standard deviation at the top. Note that the small QNR fusion protein is a significantly more potent inhibitor of FtsZ assembly than the almost full length EzrA (27–562) fusion. EzrA refers to an N-terminal Thioredoxin fusion to EzrA (27–562) with a C-terminal 6XHis tag, while QNR refers to the last 69 residues of EzrA fused in the same orientation to Thioredoxin and 6XHis. QNR(R510D) includes a mutation in the QNR motif of the 69 residue construct that disrupts EzrA localization to midcell *in vivo* [7]. Thio refers to the Thioredoxin-6XHis control protein. Bars equal standard deviation from three repeated experiments. (B) Representative trace of data from a 90°-angle light scattering reaction indicating the dose dependent nature of QNR mediated inhibition of FtsZ assembly. A.U. refers to arbitrary units.

Light scattering suggests the 69 residue QNR domain is sufficient for inhibition of FtsZ assembly (Figure 2A). In a standard assay for FtsZ assembly, 90° angle light scattering, the QNR fusion protein inhibited FtsZ assembly by ∼85%, at a 1∶1 molar ratio. This inhibition is strikingly higher than that observed with a comparable fusion to a significantly larger EzrA polypeptide that is missing only the transmembrane domain (Thio-EzrA [27–562]) (Figure 1). The larger fusion protein inhibited FtsZ assembly by ∼50% at the same molar ratio (Figure 2). We observed complete inhibition of FtsZ assembly at a 4∶1 ratio of the QNR fusion to FtsZ. A 4∶1 ratio of EzrA (27–562) to FtsZ resulted in only 70% inhibition (Figure 2). Consistent with the QNR motif playing a role in the EzrA-FtsZ interaction *in vitro*, a QNR fusion encoding the R510D mutation inhibited FtsZ assembly only 50% at a 4∶1 ratio of fusion protein to FtsZ (Figure 2). A thioredoxin control protein had no significant impact on FtsZ assembly. Specifically why the QNR domain is a more potent inhibitor of FtsZ assembly than EzrA (27–562) is unclear. Possibilities include superior solubility and/or enhanced access to the FtsZ polypeptide in the absence of bulky coiled-coil motifs. The former is consistent with previous work indicating that EzrA has a tendency to aggregate *in vitro* under the low salt conditions we routinely employ for assaying FtsZ assembly [4].

Unfortunately, we were unable to definitively test the function of the QNR domain *in vivo*. Expression of a *tm-QNR-gfp* construct consisting of EzrA's N-terminal transmembrane domain (EzrA residues 1-31), the QNR domain (EzrA residues 499–562), and a C-terminal GFP tag, from the native *ezrA* promoter failed to complement *ezrA* null mutant (Figures 1, S3A, S3B in File S1). However, the TM-QNR-GFP polypeptide was only faintly visible on a quantitative immunoblot suggesting it may be misfolded and/or degraded (Figure S2A in File S1).

### EzrA's TM domain functions independent of primary sequence

We next investigated the role of EzrA's other domains in EzrA function. Of particular interest was EzrA's N-terminal transmembrane helix. *In vitro*, this helix is dispensable for EzrA mediated inhibition of FtsZ assembly [4]. However, the TM is required for EzrA activity *in vivo*, although a TM-less version of EzrA is still capable of localizing to the medial FtsZ ring, albeit at reduced levels [4]. Work from other laboratories has implicated the TM helices of several cell division proteins in localization to the cytokinetic ring and interactions with other components of the division machinery (e.g FtsI, YneA [16], [17]). Based on these studies we wondered if EzrA's TM helix plays a specific role in mediating division inhibition or if it merely serves to concentrate EzrA at the plasma membrane where it can most effectively inhibit aberrant FtsZ assembly and maintain the dynamic nature of the medial FtsZ ring.

**Figure 3:**
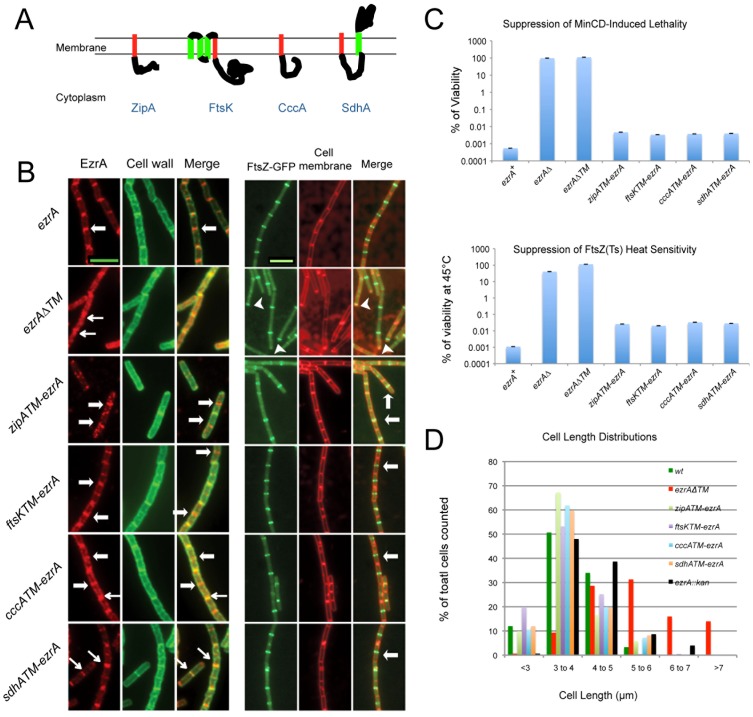
Swapping EzrA's transmembrane helix with similar domains from other proteins does not impact EzrA localization or function. (A) Cartoon of proteins used to generate EzrA TM chimeras. The TM employed in the chimera is highlighted in red. (B) (Left) Immunofluorescence images of cells expressing wild type EzrA, an EzrA deletion mutant missing the entire transmembrane domain, as well as four EzrA chimeras in which the transmembrane domain has been substituted with the appropriately oriented TM from a heterologous protein. EzrA localization is wild type in all four chimeras. (Right) Localization of FtsZ-GFP in *ezrA*-TM chimera cells. FtsZ localization is wild type in all four chimeras. Thick arrows indicate medial EzrA and FtsZ localization. Thin arrows indicate EzrA localization at septa. Arrowheads indicate polar FtsZ rings. Exposure times are equivalent for each fluorophore. Bars  = 5 µm. (C) All four chimeras behave like wild type *ezrA* alleles with regard to their sensitivity to MinCD overexpression and heat sensitivity in an *ftsZts* background. Bars equal standard deviation from three repeated experiments. (D) A plot of cell length distributions between wild type and mutant strains indicates that cell length is not impacted by the TM swaps.

To clarify the role of EzrA's TM helix in EzrA function we examined the localization and activity of four EzrA chimeras, each encoding a TM helix from a different membrane protein (Figure 3). We reasoned that if the function of EzrA's TM helix was generic (concentrating EzrA at the plasma membrane), then replacing EzrA's TM helix with that of another protein should not have any impact on EzrA function. Conversely, if EzrA's TM helix plays a role in mediating critical interactions between EzrA and other components of the division machinery, we would expect EzrA TM chimeras to exhibit defects in FtsZ localization or stability or both.

Notably, in contrast to the majority of membrane bound proteins, EzrA's orientation in the membrane is N-terminus out. We thus took special care when selecting TM helices to ensure that the final protein would be in the proper orientation (Figure 3A). In two of the chimeras, EzrA's TM helix was replaced with ones from the *E. coli* cell division proteins, ZipA and FtsK. In the other two chimeras we replaced EzrA's TM helix with ones from the *E. coli* respiratory proteins CccA and SdhA. All domain swap constructs were placed at the native *ezrA* locus under the control of *ezrA's* native promoter. A control TM deletion mutant was also expressed from the native *ezrA* locus, but for technical reasons—specifically insufficient regions of homology for recombination into the *ezrA* deletion mutant parent strain (see methods) —its expression was controlled by the IPTG inducible/repressible promoter *P_spachy_*. Quantitative immunoblotting indicated that expression of four TM chimeras was equivalent to wild type EzrA. At 1 mM IPTG, the intracellular concentration of the TM deletion mutant was ∼1.5 fold that of wild-type EzrA (Figure S1A in File S1). Membrane fractionation experiments confirmed that the TM-less EzrA was cytoplasmic, whereas wild type EzrA and the TM chimeras were concentrated in the plasma membrane (Figure S1B in File S1). EzrA was localized by immunofluorescence microscopy using antisera raised against the EzrA polypeptide [3].

In support of a model in which the primary, and potentially sole function of EzrA's TM domain is to concentrate it at the plasma membrane, all four EzrA TM chimeras were fully functional with regard to localization and inhibition of FtsZ assembly at cell poles (Figure 3B). Importantly, all TM mutants were sensitive to overexpression of the *minCD* division inhibitor and did not suppress the heat sensitivity of an *ftsZts* allele, consistent with normal medial FtsZ ring dynamics (Figure 3C). Sensitivity to *minCD* overexpression and *ftsZts* heat sensitivity is consistent with wild type EzrA function [7]. In contrast, the TM deletion mutant was phenotypically equivalent to an *ezrA* null mutant, with a very low frequency of medial localization, a high proportion of cells with at least one polar FtsZ ring, and with regard to suppression of the conditional *ftsZts* and *minCD* overexpression phenotypes (Figure 3C). Finally, the cell length distributions for all four TM chimeras were also wild type (Figure 3D). In contrast, and consistent with the loss of medial localization, *ezrA*Δ*TM* cells exhibit a wider range of cell sizes than the *ezrA* null mutant. Together these data support the idea that the primary role of the TM helix is to increase the local concentration of EzrA at the plasma membrane where it can most effectively modulate FtsZ assembly.

### EzrA's coiled-coil mutants exhibit differential localization to the FtsZ ring

A conserved and striking feature of EzrA is its extensive coiled-coil structure. Alignments of EzrAs from multiple species predict the presence of four or five long stretches of coiled-coil domains extending from shortly after the TM to just before the QNR. Because these regions are conserved at the structural rather than sequence level, similar to the TM domain, we speculated that they might play a role in either interactions between EzrA and other cell division proteins with similar motifs or in ensuring that EzrA's conformation provides optimal access to FtsZ and other components of the division machinery.

To determine the role of the coiled-coils in EzrA function, we constructed a series of deletions in each of the four predicted coiled-coils (CC) within the *B. subtilis* EzrA polypeptide (Figure 1). The location of putative coiled-coil motifs were predicted with the web based tool Paircoil2 (http://groups.csail.mit.edu/cb/paircoil2/) [18]. These mutations included single deletions of CC2 [EzrAΔ(191–353)], CC3 [EzrAΔ(376–434)], and CC4 [EzrAΔ(468–504)] as well as double deletions of CC1 and CC2 [EzrAΔ(31–353)], CC3 and CC4 [EzrAΔ(377–493)] and a triple mutant deleting the entire EzrA C-terminus including CC3, CC4, and the QNR domain [EzrAΔ(377–562)]. All deletions were constructed such that they were the only copy of EzrA in the cell and all were expressed from the native locus under the control of the native promoter. For technical reasons, including difficulty cloning certain *ezrA* constructs through *E. coli* [4], we had to rely on single crossover Campbell-like recombination into an *ezrA* deletion mutant to generate constructs at the native *ezrA* locus. We were thus unable to generate an *ezrA* construct missing only CC1 *in vivo* due to insufficient regions of homology for recombination into the *ezrA* deletion mutant parent strain (see methods). The N-terminal transmembrane domain was present in all deletion mutants.

To facilitate localization, all deletions were fused in frame to GFP. Adding GFP to EzrA's C-terminus does not alter EzrA function *in vivo*. An *ezrA*-*gfp* fusion fully complements an *ezrA* null mutant *in vivo* and exhibits wild type subcellular localization, suggesting it is fully functional *in vivo* [3], [4]. Immunoblotting with antisera against either EzrA or GFP confirmed that all fusions were properly expressed and appropriately anchored in the plasma membrane (Figure S2 in File S1).

**Figure 4:**
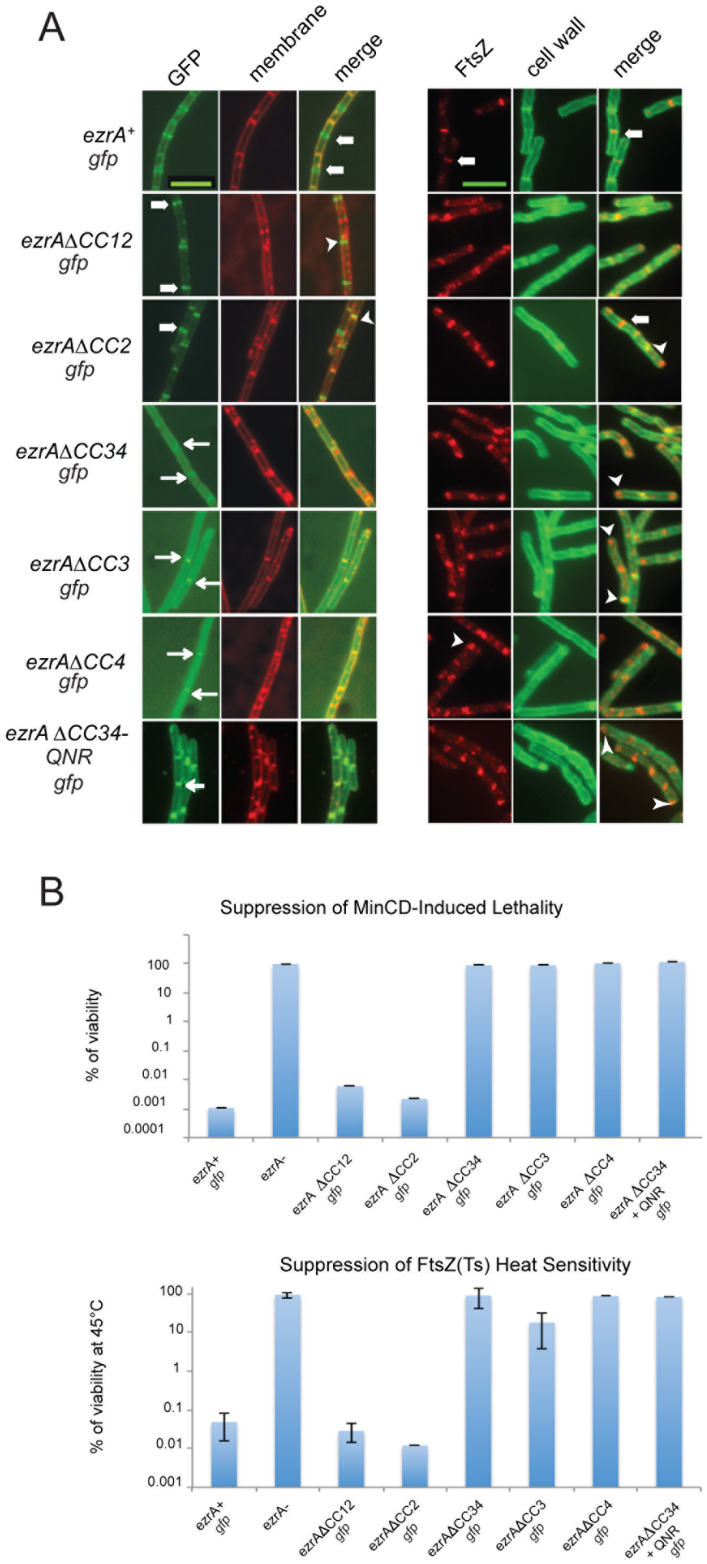
EzrA's coiled-coils exhibit separable functions *in vivo*. (A) (Left) GFP fusions to all six coiled-coil deletion mutants. Note the loss of medial EzrA localization in strains expressing GFP fusions to CC3, CC4 and QNR deletion mutants but not congenic strains expressing GFP fusions to the CC1 and CC2 deletion constructs. The EzrA CC1 and CC2 deletion mutants localize to cell poles as well as midcell, most likely through interactions with FtsZ at those subcellular positions (Right). FtsZ localization in the same strain backgrounds by immunofluorescence microscopy. Polar FtsZ rings are readily apparent in all six deletion mutants indicating that all four coiled-coils are important for EzrA mediated inhibition of polar FtsZ assembly. Thick arrows indicate medial EzrA and FtsZ localization. Thin arrows indicate EzrA localization at septa. Arrowheads indicate polar EzrA and FtsZ rings. The cell in the ΔCC2 panel is an example of cell that failed to divide and exhibits FtsZ rings at both quarter (arrow) and polar (arrowhead) positions. Exposure times are equivalent for each fluorophore. Bars  = 5 µm. (B) Consistent with loss of medial EzrA localization, deletion of EzrA CC3 and/or CC4 suppresses the lethality associated with overexpression of the MinCD division inhibitor and the heat sensitivity of the *ftsZts* allele. Defects in CC1 and CC2 are comparable to wild type *ezrA* in these assays, indicating that they are largely wild type for function at midcell. Bars equal standard error from three repeated experiments.

Visualization of mutant *ezrA* constructs by fluorescence microscopy suggests that CC1 and CC2 are largely dispensable for EzrA's interaction with the FtsZ ring, while CC3 and CC4 are essential for this activity (Figure 4A left). Deletion of either CC1 and CC2 together [*ezrAΔ(31–353)*] or CC2 alone [*ezrAΔ(191–353)*] led to an intermediate phenotype with regard to medial localization. While ∼71% (71/100) of cells expressing wild-type EzrA exhibited medial EzrA localization, cells expressing either the CC1 and CC2 double deletion or the CC2 deletion alone exhibited medial EzrA localization in only ∼49% (49/100) and ∼36% (36/100) of cells respectively (Figure 4).

In contrast, deletion of either CC3 [*ezrAΔ(376–434)*] or CC4 [*ezrAΔ(468–504)*] individually or together [*ezrAΔ(377–493*)] completely abolished medial EzrA localization, as did deletion of the entire C-terminus of EzrA including CC3, CC4, and the QNR domain [*ezrAΔ(377–562)*] consistent with previous reports (Figure 4A) [7]. Strikingly, despite clearly being retained in the membrane in fractionation experiments (Figure S2 in File S1), all of these constructs appeared to be diffusely cytoplasmic in localization with some occasional concentration observed at midcell (open arrows Figure 4A left). The lack of medial EzrA localization suggests all three constructs have lost the ability to interact with FtsZ.

**Figure 5:**
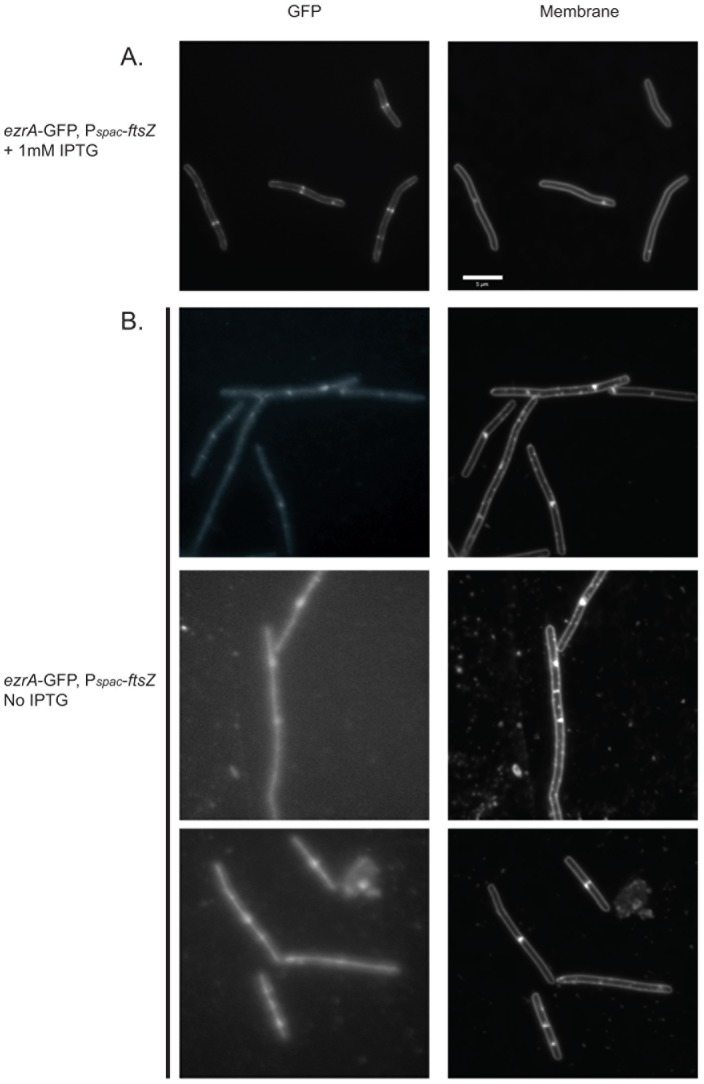
FtsZ is required for EzrA localization to the midcell. Strain PL851 encoding *ezrA*-gfp and an IPTG inducible *ftsZ* allele, *ftsZ*::P*_spac_*-*ftsZ*, was grown in LB at 37°C for three generations in the presence (A) or absence of inducer (B). Depletion of FtsZ disrupts EzrA-GFP localization to midcell. Instead, EzrA-GFP appears diffusely distributed throughout the cell. Exposure times are the same in A and B, however brightness and contrast were increased in B to allow for better visualization of diffuse EzrA-GFP localization.

Close examination of micrographs indicates that the CC1 and CC2 deletion mutants themselves localize to polar positions while CC3 and CC4 mutants did not (Figure 4A left). This finding suggests these deletion mutants retain the ability to interact with FtsZ via CC3, CC4 and the QNR domain (Figure 5). Together these data suggest that the CC1 and CC2 deletions retain the ability to interact with FtsZ, and that critical determinants for interaction with FtsZ are present in CC3 and CC4 as well as in the QNR motif as previously reported [7].

### All four coiled-coils are required to inhibit aberrant FtsZ assembly at cell poles, however, only CC3 and CC4 are required for the dynamic nature of the medial FtsZ ring

A primary function of EzrA is to inhibit aberrant FtsZ assembly and division at cell poles [3]. To determine if any of the coiled-coil deletions impact EzrA's role as spatial regulator of FtsZ assembly, we examined the pattern of FtsZ ring formation in the six coiled-coil mutant strains by immunofluorescence microscopy.

Our data suggests that all four coiled-coils are required for EzrA mediated inhibition of polar FtsZ assembly. We observed polar FtsZ assembly at frequencies equivalent to the *ezrA* null in all six coiled-coil mutant strains (Figure 4A right). The frequency of cells with at least one polar FtsZ ring was between 52% and 56% (∼150 total counted for each mutant) in the CC mutants (Table 1), a number similar to that observed in *ezrA* null mutants (∼54%) [3].

**Table 1:** The Frequency of EzrA CC deletion mutant cells with polar FtsZ rings.

Coiled-Coil Deletion Mutants	% of cells with polar FtsZ rings
*ezrA *Δ*CC2*	52% (78/150)
*ezrA *Δ*CC12*	56% (84/150)
*ezrA *Δ*CC3*	53% (79/150)
*ezrA *Δ*CC4*	55% (83/150)
*ezrA *Δ*CC34*	54% (81/150)
*ezrA *Δ*CC34-QNR*	56% (84/150)

In contrast to polar FtsZ assembly, only CC3 and CC4 appear to play a key role in modulating stability of the medial FtsZ ring (Figure 4B). To determine if any of the coiled-coil deletion mutations affected stability of the medial FtsZ ring, we tested the resistance of the various *ezrA* coiled-coil deletions expressing GFP fusions, as their only copy of *ezrA* to overexpression of the MinCD division inhibitor. While wild-type cells exhibited an ∼10^6^ fold drop in viability in the presence of excess MinCD, cells expressing the CC1 and CC2 deletion construct [*ezrAΔ(31–353)*] or the CC2 deletion construct [*ezrAΔ(191–353)*] exhibited an intermediate phenotype (∼16,000 fold and ∼44,000-fold reduction in viability respectively). Strains expressing GFP fusions to the CC3 [*ezrAΔ(376–434)*], CC4 [*ezrAΔ(468–504)*], CC3 and CC4 [*ezrAΔ(377–493)*] deletion constructs, however were fully viable in the presence of excess MinCD (Figure 4B top), a phenotype equivalent to an *ezrA* null mutant. We observed similar results with regard to suppression of *ftsZts* heat sensitivity (Figure 4B bottom). These findings are consistent with subcellular localization data suggesting deletion of CC3 and/or CC4 but not CC1 and CC2, disrupt interaction between EzrA and FtsZ at midcell (Figure 4A).

### N and C terminal coiled-coils are differentially required for EzrA mediated inhibition of FtsZ assembly *in vitro*

As a final test of domain function, we examined the ability of EzrA deletion mutants to inhibit FtsZ assembly *in vitro* using a standard 90° angle light scattering assay. All constructs included both an N-terminal thioredoxin tag (to increase solubility), followed by the truncated EzrA polypeptide, and a C-terminal 6× His tag. All thioredoxin fusions are missing the TM domains (residues 10–26).

**Figure 6:**
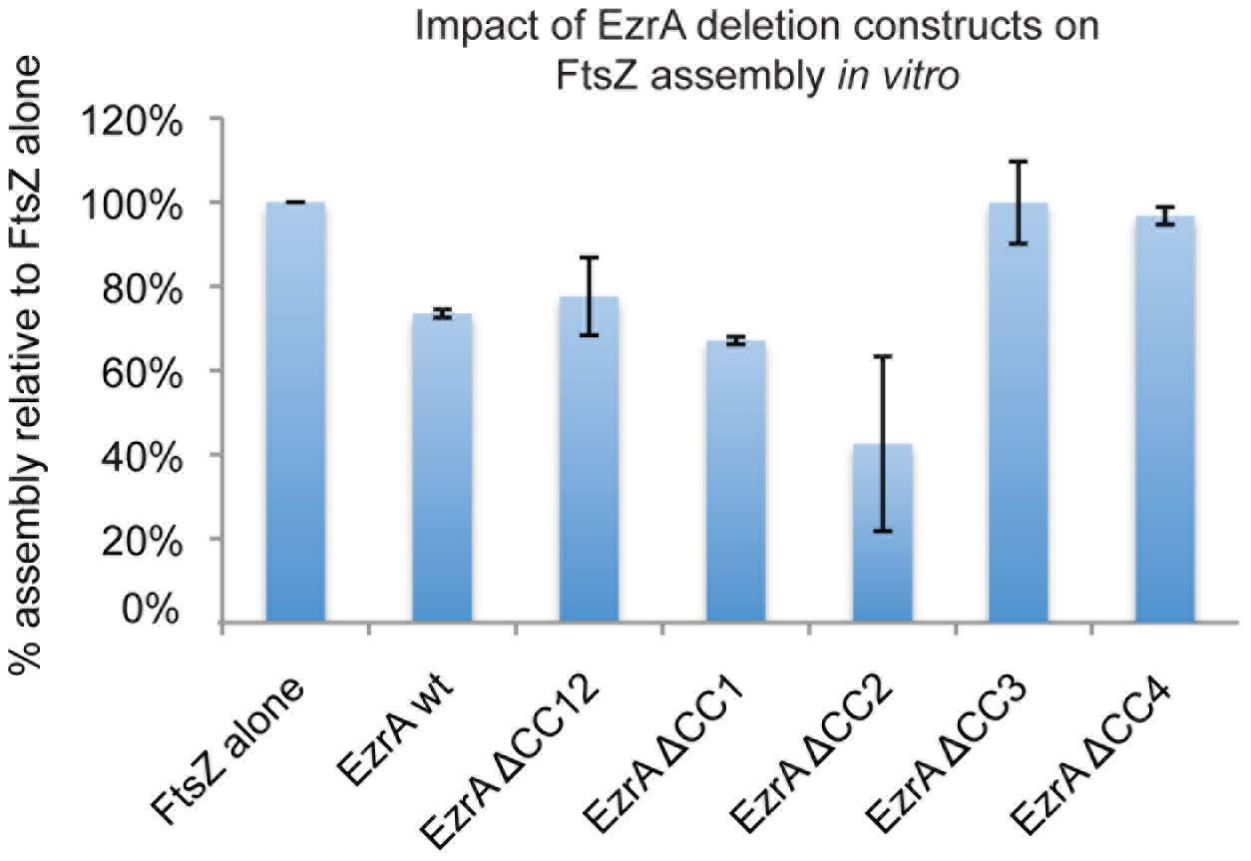
Coiled-coils 3 and 4 are required for interactions between EzrA and FtsZ *in vitro* in the context of the nearly full length EzrA (27–562). 90° angle light scattering data from FtsZ assembly reactions conducted in the presence of wild type EzrA and various coiled-coil deletion mutants. Readings were taken four times per second at 30°C, and a baseline was gathered for 1 min before the addition of 1 mM GTP to the cuvette. Baseline values are subtracted for each sample. Bars represent the maximum scatter averaged across three independent experiments with standard deviation at the top. While CC1 and CC2 are dispensable for EzrA activity *in vitro*, the loss of either CC3 or CC4 completely abolished EzrA mediated inhibition of FtsZ assembly. FtsZ is at 5 µM, EzrAs are at 10 µM, and GTP is at 1 mM in all reactions.

Consistent with our *in vivo* data, CC3 and CC4 appear to be required for EzrA mediated inhibition of FtsZ assembly *in vitro*, while CC1 and CC2 are largely dispensable for this function (Figure 6). Thioredoxin fusions to ΔCC1 [EzrAΔ(145–182)] or ΔCC2 [EzrAΔ(191–353)] inhibited FtsZ assembly by ∼40% and ∼60% respectively in a 90° angle light scattering assay, on par, or slightly better than the EzrA (27–562) fusion protein. In contrast, the ΔCC3 [EzrAΔ(376–434)] and ΔCC4 [EzrAΔ(468–504)] deletion constructs had no impact on FtsZ assembly in this assay, despite the presence of the QNR motif, suggesting these regions are required for efficient interaction between EzrA and FtsZ.

## Discussion

**Figure 7:**
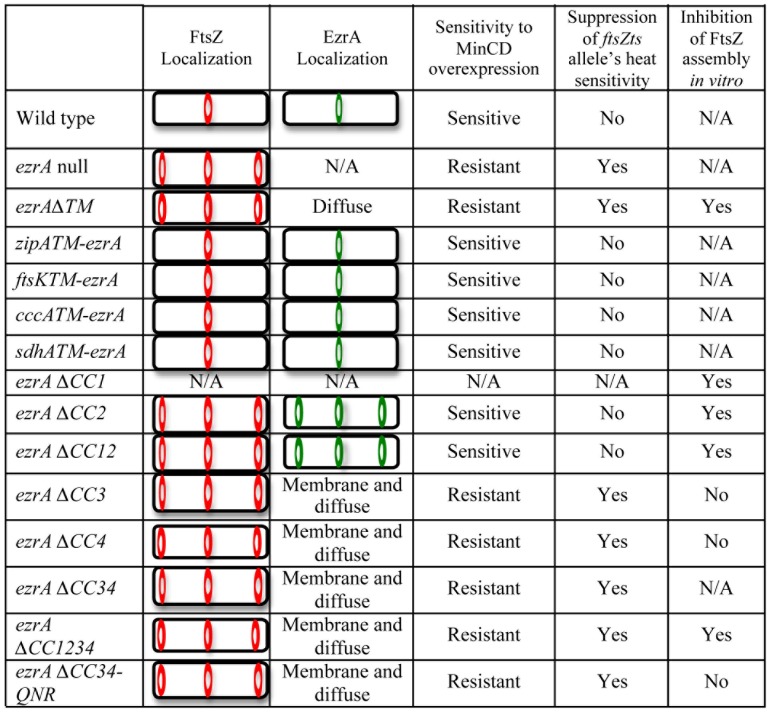
Summary of EzrA phenotypes. For comparative purposes we have summarized the phenotypes observed by each EzrA mutant used in this study. Cartoons depicting the localization of EzrA rings by fluorescence microscopy are shown in green and those depicting FtsZ rings are shown in red.

Our findings suggest that the four domains of *B. subtilis* EzrA (TM, CC12, CC34, and the QNR) have separable, yet overlapping roles in mediating EzrA's interaction with FtsZ and potentially other cellular factors. A summary of relevant phenotypes can be found in Figure 7.

In particular, our data suggest the sole function of EzrA's TM helix is to concentrate the protein at the plasma membrane where it can function most efficiently to inhibit aberrant FtsZ assembly at cell poles, promote the dynamic nature of the medial FtsZ ring, and to coordinate interactions between components of the cell division and cell wall synthesis machinery. Although deleting EzrA's TM helix entirely led to an *ezrA* null phenotype, swapping EzrA's TM helix with similarly oriented TM helices from either ZipA and FtsK, two *E. coli* cell division proteins, or TM helices from the *E. coli* respiratory proteins CccA and SdhA, had no discernable impact on EzrA function (Figure 3). All four EzrA TM chimeras exhibited wild type morphology with regard to cell size, cell division, FtsZ assembly and growth rate.

These data raise questions about physiological relevance of BACTH data suggesting EzrA interacts directly with a large number of almost exclusively extracellular proteins [8], [13]. One possibility is that such interactions are real, but dispensable for EzrA function *in vivo*. Alternatively, interaction between EzrA and primarily extracellular proteins may be artifacts of the BACTH assay itself. For example *E. coli* FtsZ may function as a bridge between EzrA and the target cell division proteins, bringing the T25 and T18 domains of adenylate cyclase into close enough proximity for synthesis of cyclic AMP. EzrA is known to inhibit assembly of *E. coli* FtsZ both *in vivo* and *in vitro* [3][PAL unpublished]. Regardless of mechanism, the apparently generic nature of EzrA's TM domain reinforces the need to obtain biochemical data confirming interactions identified between EzrA and components of the cell division machinery by BACTH.

In contrast to the TM domain, our data suggests that EzrA's four coiled-coils have specific and separable functions. CC1 and CC2 are required for EzrA mediated inhibition of FtsZ assembly at cell poles but dispensable for EzrA activity at midcell, while CC3 and CC4 appear to modulate interaction between EzrA and FtsZ throughout the cell (Figures 4 and 6). Deletion of CC1 and CC2 [EzrAΔ(31–353)] or CC2 alone [EzrAΔ(191–353)] resulted in a frequency of polar FtsZ rings approximately equivalent to that of an *ezrA* null mutant, but had little impact on EzrA localization to midcell or the stability of the medial FtsZ ring. We speculate that CC1 and CC2 play key roles in mediating interactions between EzrA and cytoplasmic components of the division machinery. For example the inability of the CC1 and CC2 deletions to inhibit polar FtsZ assembly may be due to loss of interactions between EzrA and proteins concentrated at the cell poles that normally help bring the division inhibitor into close proximity with FtsZ at this location. In contrast, *ezrA* mutants defective in CC3 or CC4 were resistant to overexpression of the *minCD* division inhibitor and also suppressed the heat sensitivity of the *ftsZts* allele, in addition to having a high frequency of polar FtsZ rings. Separable roles for CC1 and CC2 relative to CC3 and CC4 are further supported by our biochemical analysis of EzrA deletion mutants (Figure 6). *In vitro*, deletion of CC1 and CC2 in tandem or CC2 alone had little impact EzrA's ability to interact with FtsZ, while deletion of CC3 and/or CC4 completely abolished interaction between EzrA and FtsZ.

**Figure 8:**
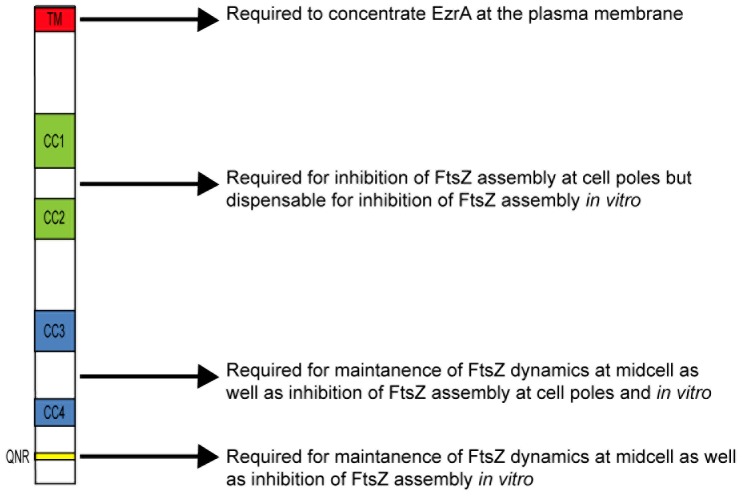
Cartoon of proposed EzrA domain function. Deletion analysis suggests EzrA has four major functional domains. The primary function of EzrA's TM domain is to concentrate EzrA at the plasma membrane where it can function most effectively to modulate FtsZ assembly at cell poles and in the cytokinetic ring. CC1 and CC2 are required for EzrA mediated inhibition of aberrant FtsZ rings at cell poles but are largely dispensable for inhibition of FtsZ assembly at midcell. CC3 or CC4 function in concert with the QNR to mediate interactions between EzrA and FtsZ throughout the cell.

Based on these data we propose that CC1 and CC2 help coordinate interactions between EzrA and FtsZ specifically at the cell poles, while the QNR domain functions together with CC3 and CC4 to inhibit FtsZ assembly through direct interactions (Figure 8). The QNR domain itself is sufficient to inhibit assembly on its own *in vitro* (Figure S3 in File S1), however CC3 and CC4 are required for inhibition of FtsZ assembly in the context of the full-length protein both *in vitro* and *in vivo*. A model in which CC3 and CC4 and the QNR interact with FtsZ independently is consistent with our finding that a mutation in the QNR motif (R510D) had little impact on interaction between EzrA and FtsZ *in vitro* in the context of the almost full length EzrA (27–562) protein [7], but completely abolished interaction between FtsZ and the QNR domain on its own (Thio-EzrA 494–562) and FtsZ (Figure S3 in File S1). Alternatively, CC3 and CC4 may play a primarily functional role, helping to bring the QNR domain into close proximity with FtsZ (Figure 8).

Intriguingly, loss of interaction with FtsZ is strongly correlated with a change in the EzrA localization pattern in live cells. In particular, those constructs that do not appear to interact strongly with FtsZ *in vivo* or *in vitro* [*ΔCC3 ezrAΔ(376–434), ΔCC4 ezrAΔ(468–504)*, ΔCC3 and ΔCC4 *ezrAΔ(377–493)*, and ΔCC3, CC4 and the QNR domain *ezrAΔ*(377*–*562)], exhibit a diffuse localization pattern that is reminiscent of cytoplasmic proteins (Figure 4A) despite being firmly anchored in the membrane according to fractionation experiments. We speculate that loss of interaction with FtsZ allows the GFP moiety to move freely in the cytoplasm at the end of what is essentially a long polypeptide tether. Consistent with this model, localization of a full-length EzrA-GFP fusion protein switched from being concentrated at the cell periphery to diffuse cytoplasmic localization upon depletion of FtsZ (Figure 5), in agreement with previous reports [3].

While our analysis has clarified the role of the various EzrA domains in EzrA function, the mechanism by which EzrA mediates inhibition of FtsZ assembly, particularly at cell poles remains elusive. Although CC1 and CC2 are clearly necessary for inhibition of aberrant FtsZ assembly at cell poles, whether or not they do so through direct interactions with FtsZ as has been suggested for the N terminus of *S. aureus EzrA* [19] or indirectly, by facilitating interactions between EzrA and other components of the cell division machinery and/or the cell wall synthesis apparatus is unclear. Structural data on EzrA's domain structure both alone and in complex with FtsZ, as well as biochemical data confirming the results of BACTH assays will go a long way towards resolving this and other questions.

## Materials and Methods

### General methods and strain construction

Strains and plasmids are listed in Tables S1 and S2 in File S1. All *B. subtilis* strains are derivatives of JH642. Standard techniques were performed on cloning and genetic manipulation. We used *E. coli* strain AG1111 [20]and TOP10 (Invitrogen) for plasmid construction, strain BB101 [21]and LMG194 (Invitrogen) for protein overexpression. *Vent* DNA polymerase (NEB) was used in PCRs. Primers of relevant constructs are listed in Table S3 in File S1, all others available upon request. Cells were grown in Luria-Bertani (LB) medium at 37°C unless otherwise noted. Strains bearing the *ftsZ-gfp* allele or *ezrA-gfp* allele were grown at 30°C. Antibiotics were used at a final concentration of ampicillin (Fisher Scientific) 100 µg/ml, chloramphenicol (Sigma) 5 µg/ml, spectinomycin (Sigma) 100 µg/ml, kanamycin (EM Sciences) 5 µg/ml, erythromycin (Fisher Scientific) 0.5 µg/ml, and lincomycin (ICM Biomedicals) 2.5 µg/ml. Isopropyl-b-D-thiogalactopyranoside (IPTG) (Sigma) was used at a concentration of 1 mM unless otherwise noted.

#### Strain construction

The *ezrA*Δ(31–353) mutation was created by deleting codons 31 through 351 of *ezrA*. The fragment of upstream *ezrA* was cloned to pJL74 [22] by *Sal*I/*Eco*RI (NEB) digestion, *ezrA* fragment of (352–562) by SpeI/SacI (NEB), and promoter and TM region by *Bam*HI/*Spe*I (NEB). The resulting plasmid pPL2609 was transformed into JH642 for and selected for *ezrA*Δ(31–351) integration at the native *ezrA* locus by double-crossover homologous recombination with spectinomycin resistance. Proper integration was verified by PCR and confirmed by sequencing. The same strategy was used on creating *ezrA*Δ(31–499) and *ezrA*Δ(191–353) mutations. C-terminal fusion with green fluorescent protein (GFP) for *ezrA*Δ(31–353), *ezrA*Δ(191–353), and *ezrA*Δ(31–499) were created by a single crossover with pPL1232, which contains a ∼900-bp 3′ *ezrA* fragment fused to *gfp* and a chloramphenicol resistant marker.

The *ezrA*Δ(376–434)-*gfp*, *ezrA*Δ(468–504)-*gfp*, and *ezrA*Δ(377–562)-*gfp* mutants were created by a single crossover recombination with pPL65 (a derivative of pUS19 containing a ∼900-bp 3′ *ezrA* fragment fused to *gfp*) [3]. The QuikChange site-directed mutagenesis kit (Stratagene) was used to construct deletion mutations in pPL65 to generate pPL65Δ(376–434), pPL65Δ(468–504) or pPL65Δ(377–562) mutation. Relevant *ezrA*-fragments were amplified and sequenced to confirm the presence of the deletion mutation before the plasmids were transformed into *B. subtilis* JH642. Recombinants were selected on spectinomycin-containing medium. Integration was confirmed by amplifying and sequencing DNA from the *ezrA* locus.

Full length EzrA and truncated versions of EzrA containing the QNR domain cannot be cloned through *E. coli*, under the control of EzrA's native promoter and, in many cases, even promoterless. EzrA is toxic to *E. coli*, apparently the result of interactions between EzrA and *E. coli* FtsZ [4]. We were only able to obtain GFP fusions to deletions of coiled-coils 2-4 by single cross-over homologous recombination of appropriate 3′ fragments of *ezrA* (e.g. one missing residues 191 to 353 for the CC2) deletion fused to GFP into an *ezrA* deletion mutant strain. FtsZ was localized by expressing a second copy of FtsZ fused to GFP from the amylase locus at a low level taking advantage of the *P_spac_* IPTG inducible promoter.

To generate the *ezrA*ΔTM allele, we amplified a TM-less *ezrA* fragment of ∼700-bp from *B. subtilis* chromosomal DNA starting from codon 22 and ligated this fragment into the pPL82 expression vector at *Sph*I/*Hin*dIII sites. pPL82 integrates into the amylase locus and drives expression in the presence of IPTG [6]. Ligation reactions were transformed directly to JH642, and single crossover recombinants were selected on chloramphenicol containing medium. The resulting strain encoding the TM-less *ezrA* allele was confirmed by amplifying and sequencing chromosomal DNA.

To create the *ezrA-TM* chimera alleles, we used two step overlapping PCR to construct chimeric *ezrA* fragments. Briefly we amplified of the Shine-Dalgarno sequence and start codon of EzrA from *B. subtilis* chromosomal DNA, a TM fragment of interest from *E. coli* chromosomal DNA, and an *ezrA* fragment that spanned codons 22 to 82. Overlapping PCR was then used to fuse all three fragments in frame and the resulting fragment was cloned into pJL74 *Eco*RI/*Hind*III. The resulting plasmid was transformed to JH642, and single crossover recombinants were selected with spectinomycin medium. All chimeras were confirmed by sequencing.

Strains used for the *minCD* and *ftsZ*(Ts) suppression assays were constructed by transforming the chromosome from the tagless *ezrA* mutant strains into the relevant background and selecting for the appropriate antibiotic resistance. *minCD* and *ftsZ*(Ts) suppression assays were conducted as previously described [7], [23], [24].

The Thio-QNR-His fusion under P*_BAD_* control was constructed by amplification of the last 69 residues of EzrA (494–562), from *B. subtilis* chromosomal DNA, followed by TOPO cloning (Invitrogen) as described previously for the wild-type EzrA fusion (Thio-EzrA-His) [25]. After sequencing verification, the plasmid was transformed into “One Shot chemically competent” TOP10 cells (Invitrogen) and then isolated and transformed into a BB101 background [21] for protein induction and purification. The Thio-QNR (R510D)-His fusion construct was created by the single mutation (R510D) using a QuikChange site-directed mutagenesis kit (Stratagene) on pPL2780 [25]. All Thio-EzrA-His CC deletion mutants were created using a QuikChange site-directed mutagenesis kit (Stratagene) on pRS3.

#### Fluorescence microscopy

An Olympus BX51 microscope equipped with an OrcaERG camera was used to capture images from both live samples and fixed cells. Openlab version 4.0 (Improvision) was used for image analysis and Adobe Photoshop CS (Adobe Systems) was used for image processing.

For EzrA-GFP fusions, live cells were visualized as described previously [26]. Briefly, cells grown to mid-exponential phase (optical density at 600 nm, ∼0.4) and were stained with the membrane dye FM 4–64 (Invitrogen) at a dilution of 1∶1,000 for about 1 min and then placed on 1% agarose in 1× phosphate-buffered saline pads on glass microscope slides. EzrA-GFP localization among strains was imaged. For FtsZ-GFP, the background strain with *P_spac_-ftsZ-gfp* allele was used. 10 µM of IPTG was added at one hour prior to imaging.

For immunofluorescence microscopy, cells were fixed by paraformaldehyde and glutaraldehyde treatment, as described previously [26]. Fixed cells were incubated with lysozyme for 5 to 10 min prior to the addition of the primary antibody. FtsZ or EzrA was detected using affinity-purified polyclonal rabbit anti-FtsZ sera or anti-EzrA sera [3], [27] in combination with donkey anti-rabbit sera conjugated to Cyanine-3 (Jackson Immunoresearch). Cell walls were visualized with wheat germ agglutinin conjugated to fluorescein (Invitrogen).

### Fractionation and quantitative immunoblotting

*B. subtilis* cultures were grown overnight at 37°C in LB broth with the appropriate antibiotics. Strains were then backdiluted 1∶100 into fresh LB broth + antibiotics and grown up to an OD_600_ of ∼0.6. Cultures were harvested by spinning at 3200×g for 10 minutes at 4°C. Pellets were then washed with 10 ml of Buffer A (0.1 M KPO_4_ {pH 7.0} 1 mM EDTA 10 mM MgCl_2_ ddH_2_O). The pellet was resuspended in 15 ml of Buffer A +50 ul of 100 mM AEBSF, 125 ul of 20 mg/ml lysozyme and incubated for 10 minutes at 37°C. Cells were lysed by French Press and extracts were transferred to 15 ml conical tubes. Extracts are centrifuged at 5000×g for 20 minutes at 4°C. Supernatant was taken and then spun again using identical conditions. The recovered supernatant was then transferred to a Beckman Coulter 70ti rotor. 10 ml of Buffer A was added to cell extracts that are then centrifuged at 50,000 rpm for 1 hour at 4°C. Following centrifugation supernatant was removed and stored as soluble fraction. Pellet is washed with 25 ml of Buffer A and spun again using identical conditions. Remaining pellet is then resuspended in 1 ml of Buffer A and was stored as the membrane fraction. Total protein was then quantified by Bradford Assay to normalize loading volumes for immunoblots. Immunoblots were performed as described previously [4].

#### Protein purification

Native *B. subtilis* FtsZ was expressed and purified as described [4]. EzrA's TM domain (residues 1–26) was removed for all *in vitro* work. EzrA (27–562) and more truncated EzrA species were cloned into a pBad/Thio-TOPO vector (Invitrogen), creating a Thioredoxin-{EzrA}-6XHis fusion protein (Thio-EzrA-His). Each construct was expressed in *E. coli* strain BB101 [21], purified by nickel affinity chromatography and coupled with gel filtration chromatography (S-300), and concentrated using an Amicon-30 filter, except for Thio-QNR-His, which was concentrated using an Amicon-10 filter. A Thioredoxin-6XHis (Thio) control protein was expressed and purified using the same strategy. Purified proteins were in the EzrA buffer 175 (50 mM HEPES [pH 7.5], 175 mM NaCl, 1 mM EGTA). Glycerol was added to a final concentration of 10%, and aliquots were flash frozen at −80°C.

#### 90° angle light scattering assay

Light-scattering assays were conducted as described in [23] using a DM-45 spectrofluorimeter (Olis). Readings were taken four times per second at 30°C, and a baseline was gathered for 1 min before the addition of 1 mM GTP to the cuvette. Baseline values are subtracted for each sample. Reactions contained 50 mM morpholinoethanesulfonic acid (MES) pH 6.5 50Mm KCl, 5 mM MgCl_2_, 1 mM EGTA, and 5 µM FtsZ. 5, 10, or 20 µM of Thio-{EzrA}-His species were used in the reaction. Thio-{EzrA}-His, or Thio, was diluted in EzrA buffer to adjust the volume before adding into the reaction. All reactions were preceded by a reference reaction containing FtsZ alone. Relative assembly was calculated by dividing the maxima for the sample reactions by the maxima for the FtsZ-only reference. Assembly values reflect an average of three experiments and error bars reflect the standard deviations.

## Supporting Information

File S1 **Figure S1**, (A) Quantitative immunoblot of lysates from wild type and *ezrA* TM mutant cells. All constructs were expressed from the native promoter with the exception of *ezrA*ΔTM, which was expressed from the IPTG inducible *Pspachy* promoter. FtsZ loading control on bottom. Primary rabbit antisera raised against EzrA or FtsZ was detected using secondary anti-rabbit serum conjugated to horse radish peroxidase. (B) Immunoblot of membrane fractionation of wild type and *ezrA* TM mutant lysates. As expected, wild type EzrA and TM helix chimeras are concentrated in the membrane fraction. The TM-less mutant is only in the cytoplasmic fraction. **Figure S2**, (A) Quantitative immunoblot of coiled-coil deletion constructs. FtsZ loading control on bottom. Note faint TM-QNR-GFP band on far right, consistent with degradation. (B) (Top three panels) Representative immunoblots of membrane fractionations from coiled-coil deletion strains. Three independent experiments are presented. Degradation over the course of the two-day assay, the latter steps of which were performed in the absence of protease inhibitors, led to variation in protein levels between experiments. Molecular weight marker is visible on far left of all three blots (whitish band). (Bottom) Soluble control (FtsZ). (C) Immunoblot of soluble fractions from coiled-coil deletion strains probed with anti-GFP sera (top) or anti-FtsZ sera (bottom). No GFP was visible in soluble fractions, consistent with membrane retention of all CC deletion mutants. Primary rabbit antibody against GFP (Genscript) or FtsZ was detected using secondary anti-rabbit serum conjugated to HRP. **Figure S3**, (A) Micrographs of GFP fusions to wild type EzrA, the full length EzrA(R510D) mutant, and an EzrA deletion mutant [*ezrAΔ(31–499)*] that includes EzrA's native transmembrane helix and QNR patch but is missing all four coiledcoils. Note absence of medial localization in both the *ezrA(R510D)* and *ezrAΔ(31–499)* images. (B) FtsZ localization by immunofluorescence microscopy. Note the presence of polar FtsZ rings in the *ezrAΔ(31–499)* images. (A and B). Thick arrows indicate medial EzrA and FtsZ localization. Thin arrows indicate EzrA localization at septa. Arrowheads indicate polar FtsZ rings. Exposure times are equivalent for each fluorophore. Bars  = 5 µm. (C) Consistent with loss of medial localization, the *ezrAΔ(31–499)* allele is equivalent to an *ezrA* null with regard to its ability to suppress the lethality associated with overexpression of the MinCD inhibitor or the heat sensitivity of the ftsZts *allele*. Bars equal standard deviation from three repeated experiments. **Table S1**, Bacterial Strains Used in this Study. **Table S2**, Plasmids used in this study. **Table S3**, Primers used in this study.
(PDF)
