# Supplementary material for: Functional Domain Analysis of the Cell Division Inhibitor EzrA
Source: PLoS One. 2014 Jul 28;9(7):e102616. doi: 10.1371/journal.pone.0102616 (PMC4113482; doi:10.1371/journal.pone.0102616)
Supplement: File S1 — Figure S1, (A) Quantitative immunoblot of lysates from wild type and ezrA TM mutant cells. All constructs were expressed from the native promoter with the exception of ezrAΔTM, which was expressed from the IPTG inducible Pspachy promoter. FtsZ loading control on bottom. Primary rabbit antisera raised against EzrA or FtsZ was detected using secondary anti-rabbit serum conjugated to horse radish peroxidase. (B) Immunoblot of membrane fractionation of wild type and ezrA TM mutant lysates. As expected, wild type EzrA and TM helix chimeras are concentrated in the membrane fraction. The TM-less mutant is only in the cytoplasmic fraction. Figure S2, (A) Quantitative immunoblot of coiled-coil deletion constructs. FtsZ loading control on bottom. Note faint TM-QNR-GFP band on far right, consistent with degradation. (B) (Top three panels) Representative immunoblots of membrane fractionations from coiled-coil deletion strains. Three independent experiments are presented. Degradation over the course of the two-day assay, the latter steps of which were performed in the absence of protease inhibitors, led to variation in protein levels between experiments. Molecular weight marker is visible on far left of all three blots (whitish band). (Bottom) Soluble control (FtsZ). (C) Immunoblot of soluble fractions from coiled-coil deletion strains probed with anti-GFP sera (top) or anti-FtsZ sera (bottom). No GFP was visible in soluble fractions, consistent with membrane retention of all CC deletion mutants. Primary rabbit antibody against GFP (Genscript) or FtsZ was detected using secondary anti-rabbit serum conjugated to HRP. Figure S3, (A) Micrographs of GFP fusions to wild type EzrA, the full length EzrA(R510D) mutant, and an EzrA deletion mutant [ezrAΔ(31–499)] that includes EzrA's native transmembrane helix and QNR patch but is missing all four coiledcoils. Note absence of medial localization in both the ezrA(R510D) and ezrAΔ(31–499) images. (B) FtsZ localization by immunofluorescence [file pone.0102616.s001.pdf]

Supporting Information

Supporting Figure Legends

Figure S1. (A) Quantitative immunoblot of lysates from wild type and *ezrA* TM mutant cells. All constructs were expressed from the native promoter with the exception of *ezrA*ΔTM, which was expressed from the IPTG inducible *Pspachy* promoter. FtsZ loading control on bottom. Primary rabbit antisera raised against EzrA or FtsZ was detected using secondary anti-rabbit serum conjugated to horse radish peroxidase. (B) Immunoblot of membrane fractionation of wild type and *ezrA* TM mutant lysates. As expected, wild type EzrA and TM helix chimeras are concentrated in the membrane fraction. The TM-less mutant is only in the cytoplasmic fraction.

Figure S2. (A) Quantitative immunoblot of coiled-coil deletion constructs. FtsZ loading control on bottom. Note faint TM-QNR-GFP band on far right, consistent with degradation. (B) (Top three panels) Representative immunoblots of membrane fractionations from coiled-coil deletion strains. Three independent experiments are presented. Degradation over the course of the two-day assay, the latter steps of which were performed in the absence of protease inhibitors, led to variation in protein levels between experiments. Molecular weight marker is visible on far left of all three blots (whitish band). (Bottom) Soluble control (FtsZ). (C) Immunoblot of soluble fractions from coiled-coil deletion strains probed with anti-GFP sera (top) or anti-FtsZ sera (bottom). No GFP was visible in soluble fractions, consistent with membrane retention of all CC deletion mutants. Primary rabbit antibody against GFP (Genscript) or FtsZ was detected using secondary anti-rabbit serum conjugated to HRP.

Figure S3. (A) Micrographs of GFP fusions to wild type EzrA, the full length EzrA(R510D) mutant, and an EzrA deletion mutant [*ezrA*Δ*(31-499)*] that includes EzrA's native transmembrane helix and QNR patch but is missing all four coiled-coils. Note absence of medial localization in both the *ezrA(R510D)* and *ezrA*Δ*(31-499)* images. (B) FtsZ localization by immunofluorescence microscopy. Note the presence of polar FtsZ rings in the *ezrA*Δ*(31-499)* images. (A and B). Thick arrows indicate medial EzrA and FtsZ localization. Thin arrows indicate EzrA localization at septa. Arrowheads indicate polar FtsZ rings. Exposure times are equivalent for each fluorophore. Bars = 5μm. (C) Consistent with loss of medial localization, the *ezrA*Δ*(31-499)* allele is equivalent to an *ezrA* null with regard to its ability to suppress the lethality associated with overexpression of the MinCD inhibitor or the heat sensitivity of the *ftsZts* allele. Bars equal standard deviation from three repeated experiments.

Supporting References

1. Jaacks KJ, Healy J, Losick R, Grossman AD (1989) Identification and characterization of genes controlled by the sporulation-regulatory gene spo0H in Bacillus subtilis. J Bacteriol 171: 4121–4129.

2. Levin PA, Kurtser IG, Grossman AD (1999) Identification and characterization of a negative regulator of FtsZ ring formation in Bacillus subtilis. Proc Natl Acad Sci USA 96: 9642–9647.

3. Levin PA, Schwartz RL, Grossman AD (2001) Polymer stability plays an important role in the positional regulation of FtsZ. J Bacteriol 183: 5449–5452.

4. Weart RB, Nakano S, Lane BE, Zuber P, Levin PA (2005) The ClpX chaperone modulates assembly of the tubulin-like protein FtsZ. Mol Microbiol 57: 238–249. doi:10.1111/j.1365-2958.2005.04673.x.

5. Haeusser DP, Garza AC, Buscher AZ, Levin PA (2007) The division inhibitor EzrA contains a seven-residue patch required for maintaining the dynamic nature of the medial FtsZ ring. J Bacteriol 189: 9001–9010. doi:10.1128/JB.01172-07.

6. Ireton K, Rudner DZ, Siranosian KJ, Grossman AD (1993) Integration of multiple developmental signals in Bacillus subtilis through the Spo0A transcription factor. Genes Dev 7: 283–294.

7. Quisel JD, Burkholder WF, Grossman AD (2001) In vivo effects of sporulation kinases on mutant Spo0A proteins in Bacillus subtilis. J Bacteriol 183: 6573–6578. doi:10.1128/JB.183.22.6573-6578.2001.

8. LeDeaux JR, Grossman AD (1995) Isolation and characterization of kinC, a gene that encodes a sensor kinase homologous to the sporulation sensor kinases KinA and KinB in Bacillus subtilis. J Bacteriol 177: 166–175.

Table S1. Bacterial Strains Used in this Study

***B. subtilis* strains**	**Genotype**	**Reference**
JH642	*B. subtilis* trpC2 pheA1	[1]
PL874	JH642 *ftsZ::ftsZ-gfp spc*	[2]
PL642	JH642 *ftsZ::ftsZ-gfp* cat	[2]
PL1138	JH642 *amyE*::P_{spachy}-*minCD* cat	[3]
PL1145	JH642 *thrC*::P_{spachy}-*minCD* MLS	[4]
PL982	JH642 *amyE*::P_{spac}-*ftsZ-gfp* cat	This work
PL851	PL847 *ezrA*-gfp, P_{spac}-*ftsZ*	[2]
PL1776	JH642 *ezrA*::*ezrA*(R510D)-*gfp* spc	[5]
PL2104	JH642 *thrC*::P_{spac}-*ftsZ-gfp* MLS	This work
PL3040	JH642 *amyE*::P_{spachy}-*ezrA*ΔTM cat	This work
PL3054	JH642 *amyE*::P_{spachy}-*ezrA*ΔTM cat, *ftsZ::ftsZ-gfp* spc	This work
PL2719	JH642 *ezrA* TM:: *cccA* TM spc	This work
PL2725	JH642 *ezrA* TM:: *cccA* TM spc, *amyE*::P_{spac}-*ftsZ-gfp* cat	This work

PL2723	JH642 *ezrA* TM:: *cccA* TM spc, *amyE*::P$_{spachy}$-*minCD* cat	This work
PL2721	JH642 *ezrA* TM:: *cccA* TM spc, *ftsZ::ftsZ-gfp* cat	This work
PL2741	JH642 *ezrA* TM:: *ftsK* TM spc	This work
PL2745	JH642 *ezrA* TM:: *ftsK* TM spc, *amyE*::P$_{spac}$-*ftsZ*-gfp cat	This work
PL2743	JH642 *ezrA* TM:: *ftsK* TM spc, *amyE*::P$_{spachy}$-*minCD* cat	This work
PL2744	JH642 *ezrA* TM:: *ftsK* TM spc, *ftsZ::ftsZ-gfp* cat	This work
PL2748	JH642 *ezrA* TM:: *sdhA* TM spc	This work
PL2751	JH642 *ezrA* TM:: *sdhA* TM spc, *amyE*::P$_{spac}$-*ftsZ-gfp* cat	This work
PL2749	JH642 *ezrA* TM:: *sdhA* TM spc, *amyE*::P$_{spachy}$-*minCD* cat	This work
PL2750	JH642 *ezrA* TM:: *sdhA* TM spc, *ftsZ::ftsZ-gfp* cat	This work
PL1548	JH642 *ezrA* TM:: *zipA* TM spc	This work
PL2692	JH642 *ezrA* TM:: *zipA* TM spc, *amyE*::P$_{spac}$-*ftsZ-gfp* cat	This work
PL2701	JH642 *ezrA* TM:: *zipA* TM spc, *amyE*::P$_{spachy}$-*minCD* cat	This work
PL2647	JH642 *ezrA* TM:: *zipA* TM spc, *ftsZ*::*ftsZ-gfp* cat	This work
PL2694	JH642 *ezrA* TM:: *zipA* TM spc, *thrC*::P$_{spac}$-*ftsZ-gfp* MLS	This work

PL3042	JH642 *ezrA*::*ezrA-gfp* spc, *amyE*::P$_{spachy}$-*minCD* cat	This work
PL3114	JH642 *ezrA*::*ezrA-gfp* spc, *ftsZ*::*ftsZ-gfp* cat	This work
PL2734	JH642 *ezrA*::*ezrA-cfp* cat, *thrC*::P$_{spac}$-*ftsZ-gfp* MLS	This work
PL2752	JH642 *ezrA*::*kan* kan, *amyE*::P$_{spac}$-*ftsZ-gfp* cat	This work
PL2533	JH642 *ezrA*::*kan* kan, *amyE*::P$_{spachy}$-*minCD* cat	This work
PL2642	JH642 *ezrA*::*kan* kan, *ftsZ*::*ftsZ-gfp* cat	This work
PL2543	JH642 *ezrA*::*kan* kan, *thrC*::P$_{spachy}$-*minCD* MLS	This work
PL2982	JH642 *ezrA*::P$_{spachy}$-*ezrA*ΔTM cat, *amyE*::P$_{spachy}$-*ezrA-spc* spc	This work
PL2700	JH642 *ezrA*::P$_{spachy}$-*ezrA*ΔTM cat, *ftsZ*::*ftsZ-gfp* spc	This work
PL2690	JH642 *ezrA*::P$_{spachy}$-*ezrA*ΔTM cat, *thrC*::P$_{spac}$-*ftsZ-gfp* MLS	This work
PL2681	JH642 *ezrA*::P$_{spachy}$-*ezrA*ΔTM cat, *thrC*::P$_{spac}$-*minCD* MLS	This work
PL2611	JH642 *ezrA*Δ(31-353) spc	This work
PL2715	JH642 *ezrA*Δ(31-353)-*gfp* spc cat	This work
PL3051	JH642 *ezrA*Δ(31-353)-*gfp* , *thrC*::P$_{spachy}$-*minCD*	This work
PL2686	JH642 *ezrA*Δ(31-353), *amyE*::P$_{spac}$-*ftsZ-gfp*	This work

PL2682	JH642 *ezrA***Δ**(31-353), *amyE*::P_{spachy}-*minCD*	This work
PL2613	JH642 *ezrA***Δ**(31-353), *ftsZ*::*ftsZ-gfp*	This work
PL2688	JH642 *ezrA***Δ**(31-353), *thrC*::P_{spac}-*ftsZ-gfp*	This work
PL2705	JH642 *ezrA***Δ**(31-499)	This work
PL2713	JH642 *ezrA***Δ**(31-499)-*gfp*	This work
PL3052	JH642 *ezrA***Δ**(31-499)-*gfp*, *thrC*::P_{spachy}-*minCD*	This work
PL2711	JH642 *ezrA***Δ**(31-499), *amyE*::P_{spac}-*ftsZ-gfp*	This work
PL2709	JH642 *ezrA***Δ**(31-499), *amyE*::P_{spachy}-*minCD*	This work
PL2707	JH642 *ezrA***Δ**(31-499), *ftsZ*::*ftsZ-gfp*	This work
PL3017	JH642 *ezrA***Δ**(191-353)	This work
PL3019	JH642 *ezrA***Δ**(191-353)-*gfp*	This work
PL3053	JH642 *ezrA***Δ**(191-353)-*gfp*, *thrC*::P_{spachy}-*minCD*	This work
PL3119	JH642 *ezrA***Δ**(191-353), *ftsZ*::*ftsZ-gfp*	This work
PL3045	JH642 *ezrA***Δ**(376-434)-*gfp*, *amyE*::P_{spachy}-*minCD*	This work
PL3116	JH642 *ezrA***Δ**(376-434)-*gfp*, *ftsZ*::*ftsZ-gfp*	This work

PL3023	JH642 *ezrA*Δ(377-493)-*gfp*	This work
PL3044	JH642 *ezrA*Δ(377-493)-*gfp*, *amyE*::P_{spachy}-*minCD*	This work
PL3120	JH642 *ezrA*Δ(377-493)-*gfp*, *ftsZ-gfp*	This work
PL2636	JH642 *ezrA*Δ(377-562)	This work
PL872	JH642 *ezrA*Δ(377-562)-*gfp*	This work
PL3043	JH642 *ezrA*Δ(377-562)-*gfp*, *amyE*::P_{spachy}-*minCD*	This work
PL3115	JH642 *ezrA*Δ(377-562)-*gfp*, *ftsZ-gfp*	This work
PL2696	JH642 *ezrA*Δ(377-562), *amyE*::P_{spac}-*ftsZ-gfp*	This work
PL2684	JH642 *ezrA*Δ(377-562), *amyE*::P_{spachy}-*minCD*	This work
PL2614	JH642 *ezrA*Δ(377-562), *ftsZ*::*ftsZ-gfp*	This work
PL2652	JH642 *ezrA*Δ(377-562), *ftsZ*::*ftsZ-gfp*	This work
PL2698	JH642 *ezrA*Δ(377-562), *thrC*::P_{spac}-*ftsZ-gfp*	This work
PL3046	JH642 *ezrA*Δ(468-504)-*gfp*, *amyE*::P_{spachy}-*minCD*	This work
PL3117	JH642 *ezrA*Δ(468-504)-*gfp*, *ftsZ-gfp*	This work
***E. coli* strains**	**Genotype**	**Reference**

AG1111	DZR200=MC1061 F'*lacIQ lacZM*15 Tn10 tet	[6]
Top10	F- *mcrA* **Δ**(*mrr-hsdRMS-mcrBC*) **φ**80lacZ**Δ**M15 **Δ***lacX74 nupG* recA1 *araD139* **Δ**(ara-leu)7697 *galE15 galK16 rpsL*(StrR) endA1 **λ**-	Invitrogen
LMG194	F- **Δ***lacX74 galE* thi *rpsL* **Δ***phoA* (Pvu II) **Δ***ara714 leu*::Tn10	Invitrogen

Table S2. Plasmids used in this study

Plasmid	Genotype	Reference
pBAD	Overexpression vector for N-terminal His thioredoxin fusion	Life Technologies
pUS19	Integrational plasmid for spectinomycin resistance for *B. subtilis*	New England Biolabs
pPL82	P_{spachy} integration vector	[7]
pJL74	Shuttle plasmid replicating in both *E. coli* and *B. subtilis*	[8]
pRS3	QuickChange Site Mutagenesis	Stratagene
pPL65	pUS19: ~900-bp 3′ *ezrA* fragment fused to *gfp*	[2]
pPL3090	pBAD/Thio-*ezra***Δ**(1-203)-*His*	This work
pPL2984	pBAD/Thio-*ezra***Δ**(191-353)-*His*	This work
pPL2829	pBAD/Thio-*ezra***Δ**(376-434)-*His*	This work
pPL2883	pBAD/Thio-*ezra***Δ**(468-504)-*His*	This work
pPL3094	pBAD/Thio-*ezra***Δ**(468-562)-*His*	This work
pPL2998	pBAD/Thio-*ezra***Δ**(501-511)-*His*	This work
pPL2780	pBAD/Thio-*ezra*(494-562)-*His* (Thio-QNR-His)	This work
pPL2784	pBAD/Thio-QNR(R510D)-*His*	This work

pPL2609	pJL74:*ezra*Δ(31-353)	This work
pPL2986	pJL74:*ezra*Δ(191-353)	This work
pPL2703	pJL74:*ezra*Δ(31-499)	This work
pPL2607	pJL74:*ezra*Δ(377-562)	This work
pPL1232	pJL74:~900-bp 3′ *ezrA* fragment fused to *gfp*	This work
pDH26	pUS19:*ezra*Δ(376-434)-*gfp*	This work
pDH27	pUS19:*ezra*Δ(468-504)-*gfp*	This work
pPL69	pUS19:*ezra*Δ(377-562)-*gfp*	This work
pPAP1	pJL74:*ezra*TM::*zipA*TM	This work
pPL2717	pJL74:*ezra*TM::*cccA*TM	This work
pPL2739	pJL74:*ezra*TM::*ftsK*TM	This work
pPL2746	pJL74:*ezra*TM::*sdhA*TM	This work
pPL3038	pPL82:P_{spachy}-*ezra*ΔTM	This work

Table S3. Primers used in this study

	**Cloning *ezrA* full length to TOPO vector**
PLO 99	ATCTACGCCGAAATCGACCGGCTG
PLO 141	AGCGGATATGTCAGCTTTGATTTTTTC
	**Cloning N-*ezrA* to TOPO vector**
PLO 99	ATCTACGCCGAAATCGACCGGCTG
PLO 147	GGCGACATGCTCTGCATCAAGC
	**Cloning *C-ezrA* to TOPO vector**
PLO 102	AGGCTGACAGCCGGTGAGCTCGGC
PLO 141	AGCGGATATGTCAGCTTTGATTTTTTC
	***ezrA* deletion**
PLO *ezrA*F1	GATAGGAAGAGTCTGTGC

PLO *ezrA*R1-BamHI	GATC GGATCC ACAAACTCCATAATGAGC
PLO *ezrA* -F2-HindIII	GATC AAGCTT TCCGCTTAGATAATCACG
PLO *ezrA*-R2	CGGCCTTGTCACATCTTG
	***ezrA* N-terminus deletion**
PLO 990 *ezrA*-F1-SalI	GATC GTCGAC GGCATAGATAGGAAGAGTCTGTGC
PLO 991 *ezrA*-R1-EcoRI	GATC GAATTC TCCAATGACAAACTCCATAATGA
PLO 992 *ezrA*-F3-SpeI-2	GATC ACTAGT AAAGCGCCTTGATGAAATTG
PLO 993 *ezrA*-R3-SacI	GATC GAGCTC TTACACTGCGGGCTCTTTTT
	***ezrA* C-terminus deletion**
PLO 994 *ezrA*-F2-SalI	GATC GTCGAC CCTGAGAGATTAAGGTAAACAGCA
PLO 995 *ezrA*-R2-EcoRI	GATC GAATTC TGAGTAGGCGACATGCTCTG

PLO 1019 *ezrA*-R2-EcoRI-stop	GATC GAATTC TGACTAGGCGACATGCTCTG
PLO 996 *ezrA*-F4-SpeI	GATC ACTAGT GCTGACATATCCGCTTAGATAATCA
PLO 997 *ezrA*-R4-SacI	GATC GAGCTC TACTCGGAGCAGCGATTACA
	***ezrA* promoter and TM**
PLO 1017 promoter+TM-F-BamHI	GATC GGATCC CGCTTTTGCATTGATGTCAC
PLO 1018 promoter+TM-R-SpeI	GATC ACTAGT TCGATTTCGGCGTAGATTTT
	***ezrA* CC2 deletion**
del498-986	TTGAAGAAGAAACAGAAGGCGGAAAAAGCGCCTTGATG
del498-986-antisense	CATCAAGGCGCTTTTTCCGCCTTCTGTTTCTTCTTCAA
	***ezrA* CC3 deletion**
PLO 419	CTTGATGCAGAGCATGTCACTAGCAACATTCCAGGC
PLO 420	GCCTGGAATGTTGCTAGTGACATGCTGTGCATCAAG

	ezrA **CC4 deletion**
PLO 421	CAACTAAACGAACTTCCAATTCAGTTCGGAAACCGG
PLO 422	CCGGTTTCCGAACTGAATTGGAAGTTCGTTTAGTTG
	ezrA **CC1 & CC2 deletion**
F-*ezrA*-SwaI	GATC ATTTAAATCACCCTGACAGATTAAGGTAAAC
R-*ezrA*-NdeI	GATC CATATGTTAAGCGGATATGTCAGCTTTG
	ezrA **CC3 & CC4 deletion**
PLO 1059 *ezrA*-F5-PstI	GATC CTGCAG CTT GTC GAG CAG GTC ATC CT
PLO 1060 *ezrA*-R5-PstI	GATC CTGCAG TGA TTA TCT AAG CGG ATA TGT CAG C
PLO 1061 *ezrA*-R5-XbaI	GATC TCTAGA TGA TTA TCT AAG CGG ATA TGT CAG C
	ezrA **TM::*zipA* TM swap**

PLO 521 P1 w/EcoRI	GATCGAATTCTCGCTTTTGCATTGATGTCACCCCATGAAAAAA ATAG
PLO 522 P2	CAGACGCAAATCCTGCATCATAATGAGCCCCCTTGCTGTTTAC CTTAATCTG
PLO 523 P3	GTAAACAGCAAGGGGGCTCATTATGATGCAGGATTTGCGTCT GATATT
PLO 524 P4	CGGCGTAGATTTTTTTCCTGCTGGTCCAGAAACCATGTACCAG TAAAGC
PLO 525 P5	GTACATGGTTTCTGGACCAGCAGGAAAAAAATCTACGCCGAA ATCGACC
PLO 526 P6 w/ XmaI	GATC CCCGGG CTAAGCGGATATGTCAGCTTTGATTTTTTCAACTG
PLO 527 P7 w/HindIII	GATC AAGCTT AACTTTCGGCATGTGGGCTGTGAC
	***ezrA* TM::*cccA* TM swap**
PLO 521 P1 w/EcoRI	GATCGAATTCTCGCTTTTGCATTGATGTCACCCCATGAAAAAA ATAG
PLO 1042 P2 *ezrA* Prom-c550-R	AATAAGCGGGTTCCATTTCATAATGAGCCCCCTTGCTGTTTAC CTTAATCTG
PLO 1043 P3 c550 TM-F	GTAAACAGCAAGGGGGCTCATTATGAAATGGAACCCGCTTATT CCATTTTTG

PLO 1044 p4 c550 TM-R	CGGCGTAGATTTTTTTCCTAAGTCCTTTTACTGATAAAAAGAAAGTTAGACC
PLO 1045 P5 N-*ezrA*-c550-F	TTTTTATCAGTAAAAGGACTTAGGAAAAAAATCTACGCCGAAATCGACC
PLO 527 P7 w/HindIII	GATC AAGCTT AACTTTCGGCATGTGGGCTGTGAC
	***ezrA* TM::*ftsK* TM swap**
PLO 521 P1 w/EcoRI	GATCGAATTCTCGCTTTTGCATTGATGTCACCCCATGAAAAAATAG
PLO 1081 P2-*ftsK*	AGAGCAGCGCAATAGTTCCCATAATGAGCCCCCTTGCTGTTTACCTTAATCTG
PLO 1082 P3-*ftsK*	TAAACAGCAAGGGGGCTCATTATGGGAACTATTGCGCTGCTCTGCGTT
PLO 1083 P4-*ftsK*	TCGGCGTAGATTTTTTTCCTGAGTTTTTCAGCAATGGTCACCCATGACCAACC
PLO 1084 P5-*ftsK*	GTGACCATTGCTGAAAAACTCAGGAAAAAAATCTACGCCGAAATCGACC
PLO 527 P7 w/HindIII	GATC AAGCTT AACTTTCGGCATGTGGGCTGTGAC
	***ezrA* TM::*sdhA* TM swap**

PLO 521 P1 w/EcoRI	GATCGAATTCTCGCTTTTGCATTGATGTCACCCCATGAAAAAAATAG
PLO 1085 P2-*sdhA*	GATAATGCTTGATTGACTCATAATGAGCCCCCTTGCTGTTTACCTTAATCTG
PLO 1086 P3-*sdhA*	TAAACAGCAAGGGGGCTCATTATGAGTCAATCAAGCATTATCGTAGTCGGCG
PLO 1087 P4-*sdhA*	TCGGCGTAGATTTTTTTCCTCGCCATTCCTGATTCCGCTGCTTTAATTGTC
PLO 1088 P5-*sdhA*	GCAGCGGAATCAGGAATGGCGAGGAAAAAAATCTACGCCGAAATCGACC
PLO 527 P7 w/HindIII	GATC AAGCTT AACTTTCGGCATGTGGGCTGTGAC
	***ezrA* TM deletion**
PLO 1113 pJQ43-F1	TGCCACCTGACGTCTAAGAA
PLO 1114 pJQ43-F2	AAGTTGGCCGCAGTGTTATC
PLO 1115 pJQ43-F2-SpeI	GATC ACTAGT AAGTTGGCCGCAGTGTTATC
PLO 669	GATCGGATCCCTAAGCGGATATGTCAGCTTTG
	**cloning *ezrA*(QNR) to TOPO vector**
PLO 1116 F5-*ezrA*	CTTGTCGAGCAGGTCATCCT

PLO 141	AGCGGATATGTCAGCTTTGATTTTTTC

Figure S1

A

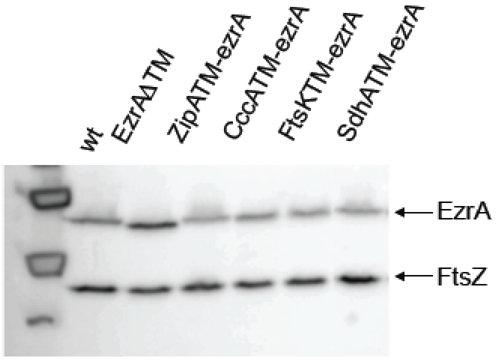
wt
EzrAΔTM
ZipATM-ezrA
CccATM-ezrA
FtsKTM-ezrA
SdhATM-ezrA
EzrA
FtsZ

B

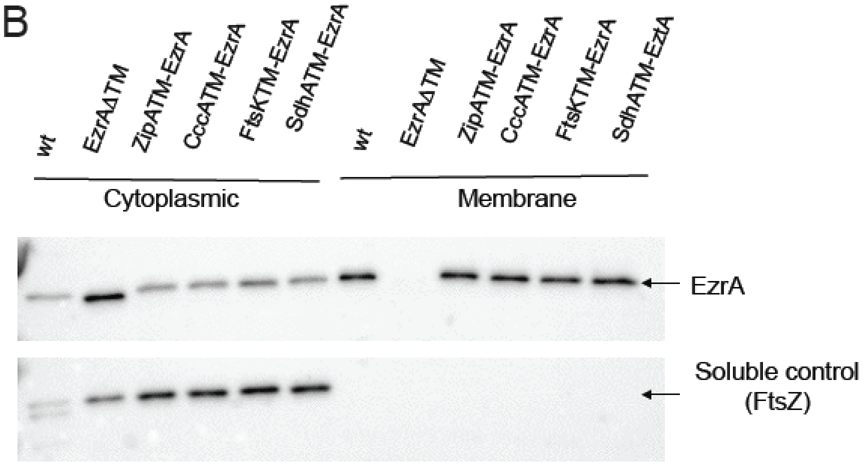
wt
EzrAΔTM
ZipATM-EzrA
CccATM-EzrA
FtsKTM-EzrA
SdhATM-EzrA
wt
EzrAΔTM
ZipATM-EzrA
CccATM-EzrA
FtsKTM-EzrA
SdhATM-EztA
Cytoplasmic
Membrane
EzrA
Soluble control
(FtsZ)

Figure S2

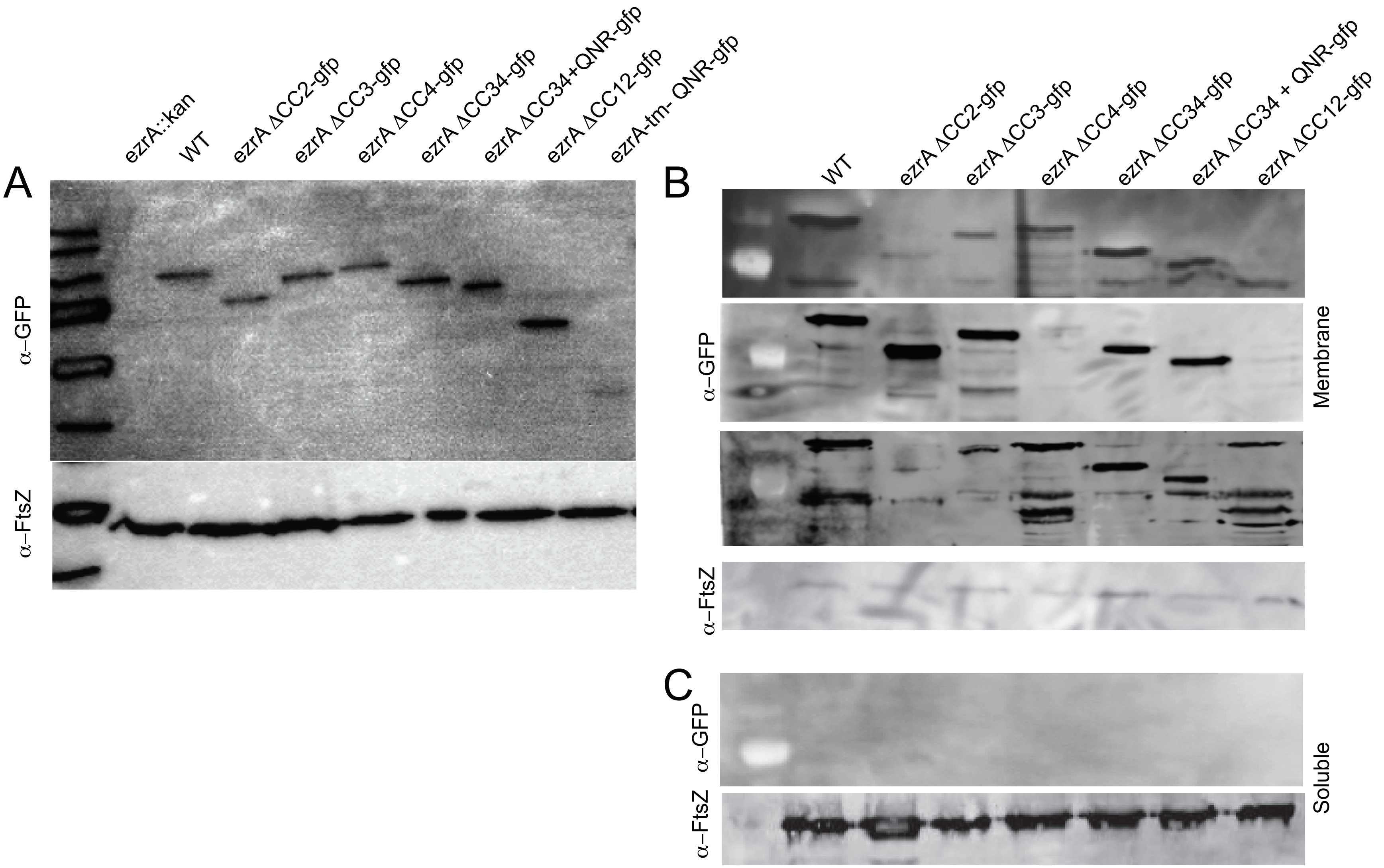
A
ezrA::kan
WT
ezrA ΔCC2-gfp
ezrA ΔCC3-gfp
ezrA ΔCC4-gfp
ezrA ΔCC34-gfp
ezrA ΔCC34+QNR-gfp
ezrA ΔCC12-gfp
ezrA-tm- QNR-gfp
α−GFP
α−FtsZ
B
WT
ezrA ΔCC2-gfp
ezrA ΔCC3-gfp
ezrA ΔCC4-gfp
ezrA ΔCC34-gfp
ezrA ΔCC34 + QNR-gfp
ezrA ΔCC12-gfp
α−GFP
Membrane
α−FtsZ
C
α−GFP
α−FtsZ
Soluble

Figure S3

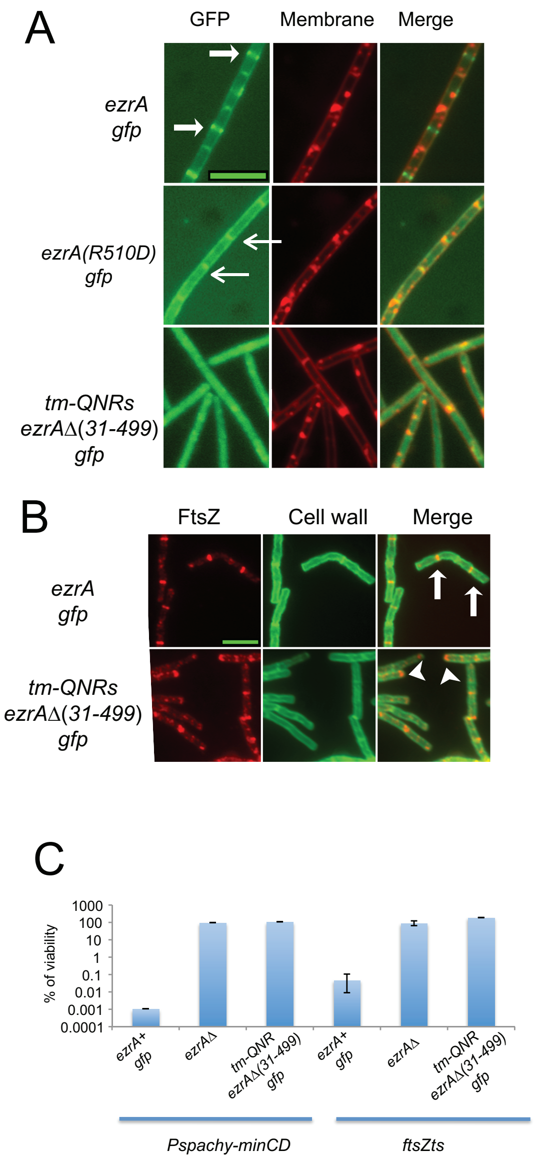
A
GFP
Membrane
Merge
ezrA
gfp
ezrA(R510D)
gfp
tm-QNRs
ezrAΔ(31-499)
gfp
B
FtsZ
Cell wall
Merge
ezrA
gfp
tm-QNRs
ezrAΔ(31-499)
gfp
C
% of viability
1000
100
10
1
0.1
0.01
0.001
0.0001
ezrA+
gfp
ezrAΔ
tm-QNR
ezrAΔ(31-499)
gfp
ezrA+
gfp
ezrAΔ
tm-QNR
ezrAΔ(31-499)
gfp
Pspachy-minCD
ftsZts
